# Boosting with recombinant MVA expressing *M. tuberculosis* α-crystallin antigen augments the protection imparted by BCG against tuberculosis in guinea pigs

**DOI:** 10.1038/s41598-017-17587-5

**Published:** 2017-12-11

**Authors:** Prachi Nangpal, Ritika Kar Bahal, Anil K. Tyagi

**Affiliations:** 1 Department of Biochemistry, University of Delhi South Campus, Benito Juarez Road, New Delhi, India; 2 Guru Gobind Singh Indraprastha University, Sector 16-C, Dwarka, New Delhi India

## Abstract

Tuberculosis (TB) is one of the major causes of mortality all over the globe. BCG, the only vaccine available against this disease has been successful in preventing the severe forms of childhood TB. However, the unsatisfactory performance of BCG in controlling the adult pulmonary tuberculosis has made the development of an effective vaccine against *M. tuberculosis* a prime objective of the TB research. In this study, a genetically stable, marker-free recombinant MVA expressing α-crystallin of *M. tuberculosis* (rMVA.*acr*) was generated which was further evaluated for its ability to impart protection as a booster vaccine against tuberculosis in a heterologous prime boost approach. Our results demonstrated that intradermal delivery of rMVA.*acr* was able to efficiently boost the BCG induced protection against *M. tuberculosis* infection in guinea pigs by significantly reducing the pulmonary bacillary load (1.27 log_10_ fewer bacilli) in comparison to BCG vaccination alone. In addition, boosting BCG vaccinated animals with intramuscular delivery of rMVA.*acr* resulted in significantly superior protective efficacy in both lungs and spleen with 0.83 log_10_ and 0.74 log_10_ CFU fewer bacilli, respectively, when compared to animals vaccinated with BCG only. These findings establish the promise of this prime-boost strategy involving rMVA.*acr* in enhancing the efficacy of BCG.

## Introduction

TB is one of the leading causes of death claiming a loss of 1.8 million human lives globally as reported by WHO in 2015^1^. One third of the world’s population is infected latently with *M. tuberculosis* and is potentially at the risk of developing an active disease^1,2^. The only preventive measure available is *Mycobacterium bovis* Bacillus Calmette-Guerin (BCG), which has fared extremely well in providing protection against childhood TB^3^. However, its efficacy in adults remains inconsistent underlying the urgency for innovative research to develop more effective vaccines against this resilient pathogen. Considering the fact that majority of the world’s population is vaccinated with BCG and this will continue in the foreseeable future, finding individuals who have not been immunized with BCG will be a difficult task for testing any new vaccines. Moreover, it is believed that finding a new replacement vaccine for BCG is a great challenge, hence, heterologous boosting involving BCG priming and boosting with another vector expressing immunodominant *M. tuberculosis* antigen appears to be a more logical approach^4–6^.

HspX or α-crystallin, a 16 kDa protein, is one of the dominant antigens expressed during the latent stages of *M. tuberculosis* infection and under various other conditions like low oxygen tension, nutrient starvation or hypoxia^7,8^. α-crystallin is a member of the small heat shock protein family, which helps in maintaining and thickening of the cell wall and in providing stability to proteins that allow the bacteria to survive under harsher conditions^7^. Besides, an increase in T cell responses are observed against HspX in healthy latent individuals as compared to active TB patients suggesting its role in maintaining a disease free state in these subjects^9^. Also, it is known that BCG does not induce potent IFN-γ responses against α-crystallin, which may suggest the failure of BCG in preventing reactivation TB^10,11^. Hence, boosting BCG with latency associated antigens like α-crystallin may prove to be an effective strategy for controlling TB.

We have earlier reported the importance of α–crystallin as a promising antigen for TB vaccine development^12–14^. In one of the study from our laboratory, we have shown that a DNA vaccine expressing α-crystallin provides considerable protection to guinea pigs against *M. tuberculosis* infection^12^. In addition, a previous study from our laboratory based on BCG priming followed by boosting with the DNA vaccine expressing α–crystallin imparted markedly better protection against *M. tuberculosis* in comparison to BCG in both guinea pigs and mice^13^. Moreover, DNA vaccine expressing α-crystallin when administered as an adjunct to standard chemotherapeutic drugs significantly delayed the reactivation TB in mice in comparison to chemotherapy alone, thus, emphasizing the immunotherapeutic potential of DNA.acr in shortening the duration of TB chemotherapy^14^. However, in the light of the fact that DNA vaccines have not shown promising results in humans in spite of their remarkable success in animal models^15^, other approaches such as viral vectored systems should be employed for the purpose of boosting and evaluated in animal models before moving to clinical studies.

Attenuated strains of vaccinia virus especially Modified Vaccinia Ankara (MVA) based systems have shown tremendous success as a potent antigen delivery agent against many infectious diseases^16^. In particular, MVA is known to be highly immunogenic and has shown promising results against diseases like tuberculosis, malaria and HIV^17–19^. Vaccination with MVA in mice has been shown to result in Th1-type cytokine response with increase in the pro-inflammatory cytokines like IL-6 and TNF-α^20^. In addition, it was shown that MVA lacks several immunomodulatory soluble proteins that bind IFN-γ, IFN-α/β, TNF and CC chemokines, thus, providing a plausible explanation for the good immunogenicity of MVA despite its poor replication in mammals^21^. Moreover, MVA has been employed for developing TB booster vaccines and has shown promising results in various animal models^22,23^. MVA.85 A, a recombinant modified vaccinia virus expressing antigen 85 A of *M. tuberculosis*, when administered to BCG primed mice resulted in superior protection than BCG alone against tuberculosis^22^. In addition, boosting BCG primed guinea pigs with MVA.85 A augmented the protective efficacy of BCG against *M. tuberculosis* ^19^. Based on several lines of evidence highlighting the potential use of MVA as an antigen delivery system, in this study, we have employed MVA virus for developing a TB booster vaccine.

In this study, we have constructed and characterized a recombinant MVA expressing α-crystallin gene of *M. tuberculosis* (rMVA.*acr*). Further, we have evaluated the protective efficacy of rMVA.*acr* for its ability to boost the efficacy of BCG in a heterologous prime-boost approach against TB in guinea pigs against *M. tuberculosis* challenge at various viral doses administered via different routes of immunization (intradermal, intramuscular and intranasal).

## Results

### Construction of plasmid transfer vector pSC65.*gfp.acr*.DR

The plasmid transfer vector called pSC65.*gfp.acr*.DR was redesigned from pSC65 which was kindly provided by Dr. Sekhar Chakrabarti (NICED, Kolkata, India)^24^. TKL and TKR in pSC65 represent the left and right region of thymidine kinase gene, which allows the vector to recombine with the thymidine kinase locus of the MVA genome. The vector contains two vaccinia virus specific promoters: P_e/l_ and P_7.5_. In pSC65, LacZ is expressed under the transcriptional control of P_7.5_ and the gene of interest can be cloned at the multiple cloning site under the control of P_e/l_. The marker system was changed from LacZ to GFP since it is easier to handle and has less technical difficulties. Hence, for this*, lac*Z gene was excised out and *gfp* gene was cloned. 0.8 kb digestion product confirmed the cloning of *gfp* gene (Supplementary Fig. S1). Site directed mutagenesis with Spe I-F and Spe I-R primers was carried to create a *Spe*I site in between the two promoters (P_e/l_ and P_7.5_) to clone a direct repeat sequence (Table 1). A fall out at 0.9 kb confirmed the presence of *Spe*I site (Supplementary Fig. S3a). Further, α-crystallin gene of *M. tuberculosis* was cloned under P_e/l_ promoter. A 0.45 kb fall out confirmed the presence and orientation of α-crystallin gene (Supplementary Fig. S3b). Next, a direct repeat sequence (amplified from the TKR region of pSC65.*gfp*.*acr*) of 260 bps was cloned between the two promoters which would help in the excision of the *gfp* gene via homologous recombination (Supplementary Fig. S5). The orientation of the direct repeat was verified by sequencing. The final construct pSC65.*gfp*.*acr*.DR was confirmed by DNA sequencing before proceeding for transfection.

**Table 1:** Primers that were employed in this study.

Aim of the PCR	Primer Sequence	Expected Amplicon size	
		MVA-WT	rMVA
To create the *SpeI* site for cloning the direct repeat in the plasmid	SpeI-F 5′GCTTTCACTAGTTCCAAACCCAC 3′		
	SpeI-R 5′GGGTGGGTTTGGAACTAGTGAAAGC 3′		
To amplify the direct repeat sequence	DR-F 5′GGATACACTAGTTTCTGTCAGCGTATGGC 3′	0.26 kb	
	DR-R 5′ GATCACTAGTGAGTCGATGTAACAC 3′		
To detect the presence of the desired recombinant (rMVA.*gfp.acr*)	TKL-F 5′ TTCTCCGTGATAGGTATC 3′		1.7 kb
	GFP-R 5′ ATCGGGATCCATAAAAATCATCACTTGTACAGCTCGTCCATGC 3′		
To confirm site-specific recombination in rMVA.*gfp.acr*	Gen-F, 5′ CTGAATTAAATATGTTCTTCATGCC 3′		1.42 kb
	Acr-R 5′ ATGGCCACCACCCTTCCC 3′		
To confirm elimination of MVA-WT in rMVA.*gfp.acr* lysate	TKL-F, 5′ TTCTCCGTGATAGGTATC 3′	0.3 kb	0.9 kb/2.0 kb
	TKR-R, 5′ GATCACTAGTGAGTCGATGTAACAC 3′		
To confirm isolation of a markerless rMVA.*acr*	Gen-F, 5′ CTGAATTAAATATGTTCTTCATGCC 3′	2.0 kb	2.58 kb
	Gen-R, 5′ CGGTCCTCAGCATAAAGTAC 3′		
*gfp* specific primers	GFP-F 5′ ATCGCTCGAGATGGTGAGCAAGGGCGAGGAG 3′		0.8 kb
	GFP-R 5′ ATCGGGATCCATAAAAATCATCACTTGTACAGCTCGTCCATGC 3′		

### Generation of recombinant MVA expressing α-crystallin gene of *M. tuberculosis*

**Figure 1:**
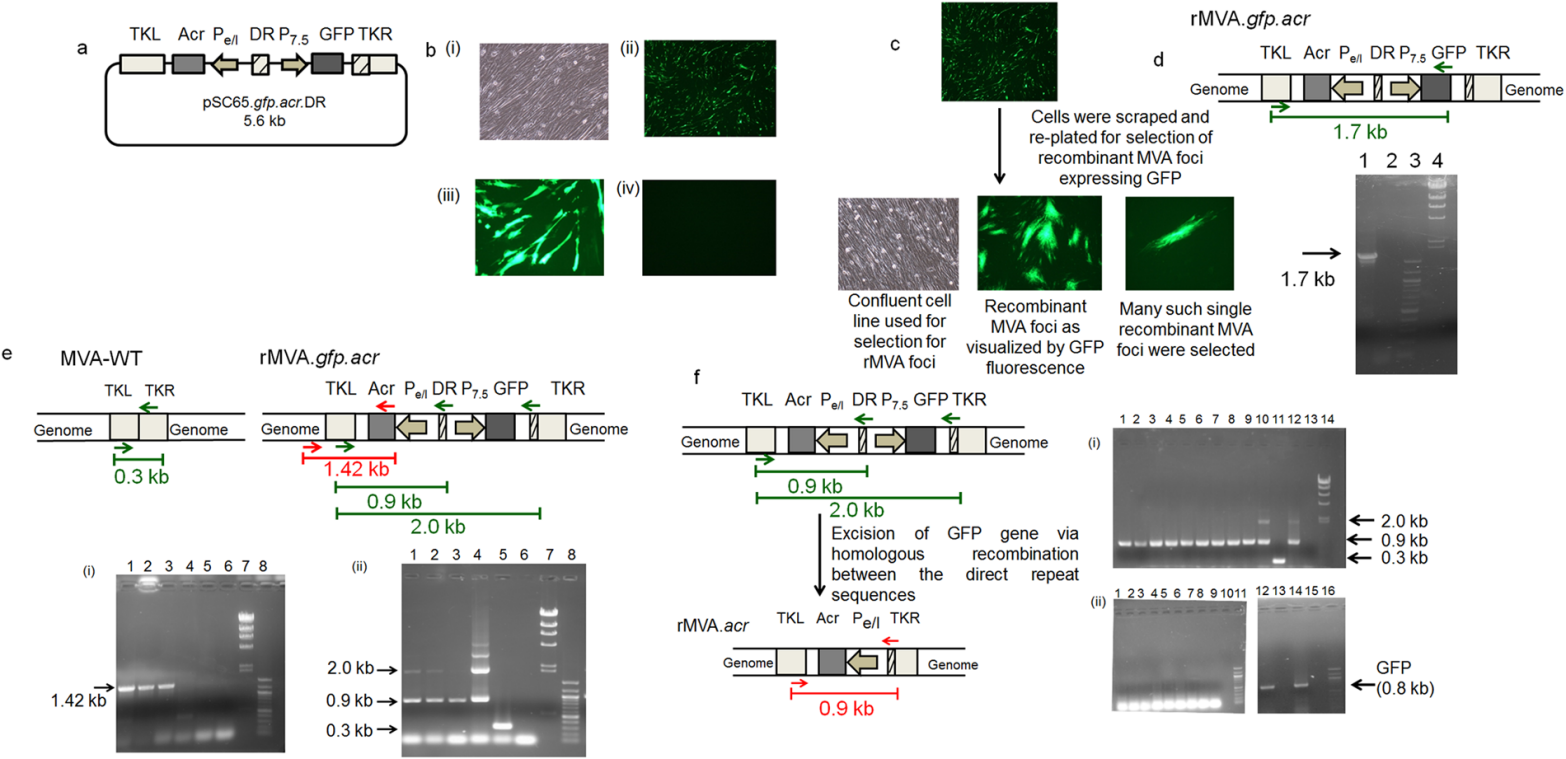
Construction of rMVA.*acr*. (**a**) Schematic diagram of the plasmid employed for the generation of recombinant MVA (rMVA.*acr*). (**b**) (i) Primary cell line used for transfection. (ii) Positive transfection of the MVA-WT infected cells with the plasmid (4X). (iii) 20X magnification of the positively transfected MVA-WT infected cells. (iv) Negative control: Cells infected with MVA-WT and lipofectamine reagent. (**c**) Selection of many individual GFP expressing recombinant foci. (**d**) Screening for the recombinant by PCR. Lane 1: rMVA.*gfp.acr*, Lane 2: MVA.WT, Lane 3: 100 bp marker and Lane 4: λ HindIII marker. The cropped gel is used in the figure, and full-length gel is presented in Supplementary Fig. S7. (**e**) (i) 1.42 kb band was observed in case of rMVA*.gfp.acr* while no band was observed when the wild type and plasmid DNA was used as a template. Lane 1–3: Three individual clones representing rMVA.*gfp.acr*, Lane 4: MVA-WT, Lane 5: Plasmid, Lane 6: Negative control, Lane 7: λ HindIII marker and Lane 8: 100 bp marker. The cropped gel is used in the figure, and full-length gel is presented in Supplementary Fig. S8. (ii) The figure shows the 2.0 kb and 0.88 kb in that DNA isolated from the three individual recombinant clones indicating that the clones are devoid of wild type MVA virus. Lane 1–3: Three individual clones representing rMVA.*gfp.acr*, Lane 4: Plasmid, Lane 5: MVA-WT, Lane 6: Negative control, Lane 7: λ HindIII marker and Lane 8: 100 bp marker. The cropped gel is used in the figure, and full-length gel is presented in Supplementary Fig. S9. (**f**) The figure shows the confirmation of marker-less recombinant MVA. (i) The gel figure shows the absence of 2.0 kb band in the nine clones selected by using limited dilution method. Lane 1–9: Nine individual clones representing marker-less rMVA.*acr*, Lane 10: rMVA.*gfp.acr*, Lane 11: MVA-WT, Lane 12: Plasmid Vector, Lane 13: Empty lane, Lane 14: λ HindIII marker. The cropped gel is used in the figure, and full-length gel is presented in Supplementary Fig. S10. (ii) The figure confirms the loss of *gfp* gene as none of the nine clones selected by using limited dilution method showed 0.8 kb amplification product thereby confirming the excision of the *gfp* gene. Lane 1–9: Nine individual clones representing marker-less rMVA.*acr*, Lane 10: empty lane, Lane 11: 100 bp marker, Lane 12: rMVA.*gfp.acr*, Lane 13: Empty lane, Lane 14: Plasmid Vector, Lane 15: empty lane and Lane 16: 100 bp marker. The cropped gel is used in the figure, and full-length gel is presented in Supplementary Fig. S11.

One of the most common strategies employed for the development of recombinant MVA is “marker transfer technique” which facilitates the insertion of gene of interest into the non-essential region such as thymidine kinase (TK) gene of the MVA genome^25,26^. The plasmid transfer vector pSC65.*gfp*.*acr*.DR was employed for transfection of MVA (MVA-1974 NIH/clone1) infected primary CEF cell line to generate recombinant MVA (Fig. 1a). The transfected cells showing GFP fluorescence were further employed to select recombinant MVA expressing GFP as described in methods (Fig. 1b and c). The presence of the 1.7 kb band obtained with recombinant MVA DNA, but not in DNA isolated from MVA-WT confirmed the selection of the recombinant (Fig. 1d).

The recombinant virus obtained above was further passaged seven times on CEF cell line to clonally purify the virus to eliminate any wild type virus population as described in methods. PCR analysis was carried out to confirm the elimination of the wild type virus. Figure 1e represents the PCR that was set up from the virus obtained after the seventh passage. Presence of 1.42 kb amplicon that was obtained by using Gen-F and Acr-R primers,confirmed site specific recombination of α-crystallin gene (Fig. 1e, Table 1). The 1.42 kb band was also sequenced thus confirming the site specific recombination of α-crystallin gene. In addition, PCR was carried to confirm the elimination of MVA-WT from the recombinant clone that was passaged for 7 times on 1° CEF cell line (Fig. 1e). For this, primers (TKL-F and TKR-R) were designed to selectively amplify region from the TKL to TKR (Fig. 1e, Table 1). Presence of 2.0 kb and 0.9 kb band in the recombinant virus and absence of 0.3 kb band (characteristic of MVA-WT), confirmed that recombinant MVA has been clonally purified and is devoid of any parental strain (Fig. 1e). One of the clonally purified recombinant virus (rMVA.*gfp.acr*) was further employed for downstream work.

Following the selection of rMVA.*gfp.acr*, next step was to remove the marker gene (*gfp*) via direct repeat excision. It is neither advisable nor permissible to use selection marker genes in the recombinant vaccines meant for human use. Hence, to isolate the rMVA which has lost selection marker *gfp* (rMVA.*acr*), limiting dilution method was employed as described in methods section. Different sets of PCR were carried out to confirm the loss of *gfp* gene. First set of PCR was carried out by using the forward primer (TKL-F) and the reverse primer (TKR-R) that binds to the repeat region, a part of TKR (Fig. 1f, Table 1). Virus isolated from all the nine wells gave a band at 0.9 kb corresponding to rMVA.*acr* whereas none of them gave a band at 2.0 kb indicating the removal of *gfp* from the recombinant MVA clones. Another PCR was set up by using GFP-F and GFP-R primers specific for *gfp* to confirm the loss of *gfp* gene (Table 1). Neither of the clones showed an amplification product of 0.8 kb corresponding to the *gfp* gene, thereby confirming the excision of *gfp* and generation of marker-less recombinant virus (rMVA.*acr*) (Fig. 1f).

### Characterization of the marker-less recombinant MVA (rMVA.*acr*)

**Figure 2:**
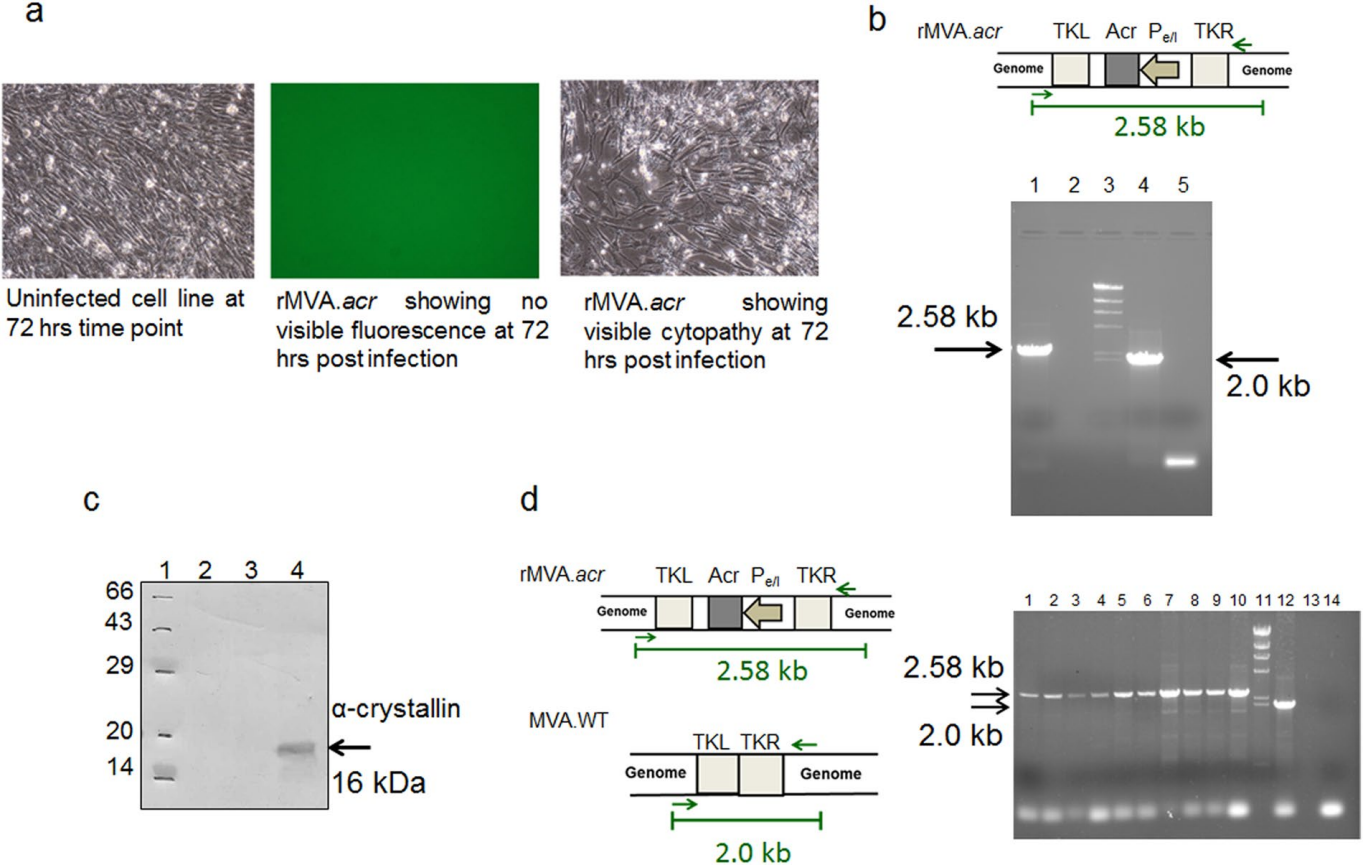
Characterization of marker free rMVA.*acr*. (**a**) The marker free rMVA.*acr* was propagated on primary CEF cell line and the clone did not show any visible fluorescence at 72 hours post infection while it induced cytopathy at 72 hours post infection. (**b**) PCR analysis of the vaccine strain was carried out by using genome primers. 2.58 kb band represents the maker-less rMVA.*acr*. Lane 1: rMVA.*acr* vaccine strain, Lane 2: Empty Lane, Lane 3: λ HindIII marker, Lane 4: MVA-WT and Lane 5: NTC: Negative control without DNA template. The cropped gel is used in the figure, and full-length gel is presented in Supplementary Fig. S12. (**c**) The immunoblot confirms the expression of α–crystallin protein from rMVA.*acr* infected CEF cell line. 50 µg of protein lysate was subjected to 15% SDS-PAGE followed by immunoblotting with monoclonal antibody against α–crystallin. Lane 1: Protein marker, Lane 2: Lysate prepared from uninfected CEF cell line, Lane 3: Lysate prepared from CEF cell line infected with MVA-WT and Lane 4: Lysate prepared from CEF cell line infected with rMVA.*acr*. The cropped blot is used in the figure, and full-length blot is presented in Supplementary Fig. S13. (**d**) Confirmation of genetic stability of rMVA.*acr* by PCR analysis. The gel picture depicts the PCR that was carried out by employing primers specific to the genomic region of the virus. A 2.58 kb amplification product was obtained with nine independent rMVA clones after 8th passage whereas a 2.0 kb amplification product was obtained with WT-MVA DNA as template. Lane 1–9: Nine independent clones of rMVA.*acr*, Lane 10: rMVA.*acr* vaccine strain, Lane 11: λ HindIII marker and Lane 12: MVA-WT, Lane13: Empty lane and Lane 14: Negative control without the DNA template. The cropped gels are used in the figure, and full-length gels are presented in Supplementary Fig. S14.

When one of the nine clones was propagated on primary CEF cell line, it showed no visible fluorescence during the course of infection thereby confirming the loss of *gfp* from the recombinant clone (Fig. 2a). We further characterized the rMVA.*acr* by PCR analysis by using Gen-F and Gen-R primers (Table 1). A 2.58 kb band confirmed the genetic integrity of the rMVA.*acr* (Fig. 2b).

Next, the expression of α-crystallin antigen by rMVA.*acr* was analyzed by immunoblotting. CEF cell lines were infected with rMVA.*acr* at a MOI of 10. The cells were harvested at 48 hours post infection and were immunoblotted with antibodies against α-crystallin. The presence of 16 kDa band confirmed the expression of α-crystallin by rMVA.*acr* (Fig. 2c).

Further, the genetic stability of the recombinant virus is a major concern for viral vector based vaccines intended for clinical investigations, because they would be amplified multiple times to reach the scale needed for manufacturing according to GMP regulations. The genetic stability of rMVA.*acr* was analyzed by passaging nine independent clones isolated from low passage stock for eight sequential passages on CEF cell line. The stability of these clones was assessed by PCR analysis by using Gen-F and Gen-R primers (Table 1). Presence of 2.58 kb amplification product observed when the DNA template was isolated from nine independent rMVA.*acr* clones (after 8 passage) confirmed the absence of any reversion to the wild type form since DNA isolated from the wild type would result in an amplification product of 2.0 kb (Fig. 2d). Further, the genetic stability of rMVA.*acr* was also assessed by immunostaining. Various dilutions of five out of the nine clones were used to infect CEF cell lines and were further subjected to immunostaining. The ratio of rMVA.*acr* and MVA-WT was assessed by immunostaining with antibodies against α-crystallin and vaccinia virus (VV). Since, the number of focuses stained with antibodies against α-crystallin and VV were same, it can be inferred that there is no reversion of recombinants back to wild type ascertaining that rMVA.*acr* is genetically stable (Table 2).

**Table 2:** Genetic stability studies of rMVA.*acr* as assessed by immunostaining.

Independent clones of rMVA.*acr*	Total no. of α-crystallin stained foci	Total no. of V.V stained foci
Clone 1	50	49
Clone 2	52	50
Clone 3	97	99
Clone 4	78	79
Clone 5	65	64

### Boosting BCG vaccinated guinea pigs with intradermal administration of rMVA.*acr* reduces the lung bacillary load following *M. tuberculosis* challenge

**Figure 3:**
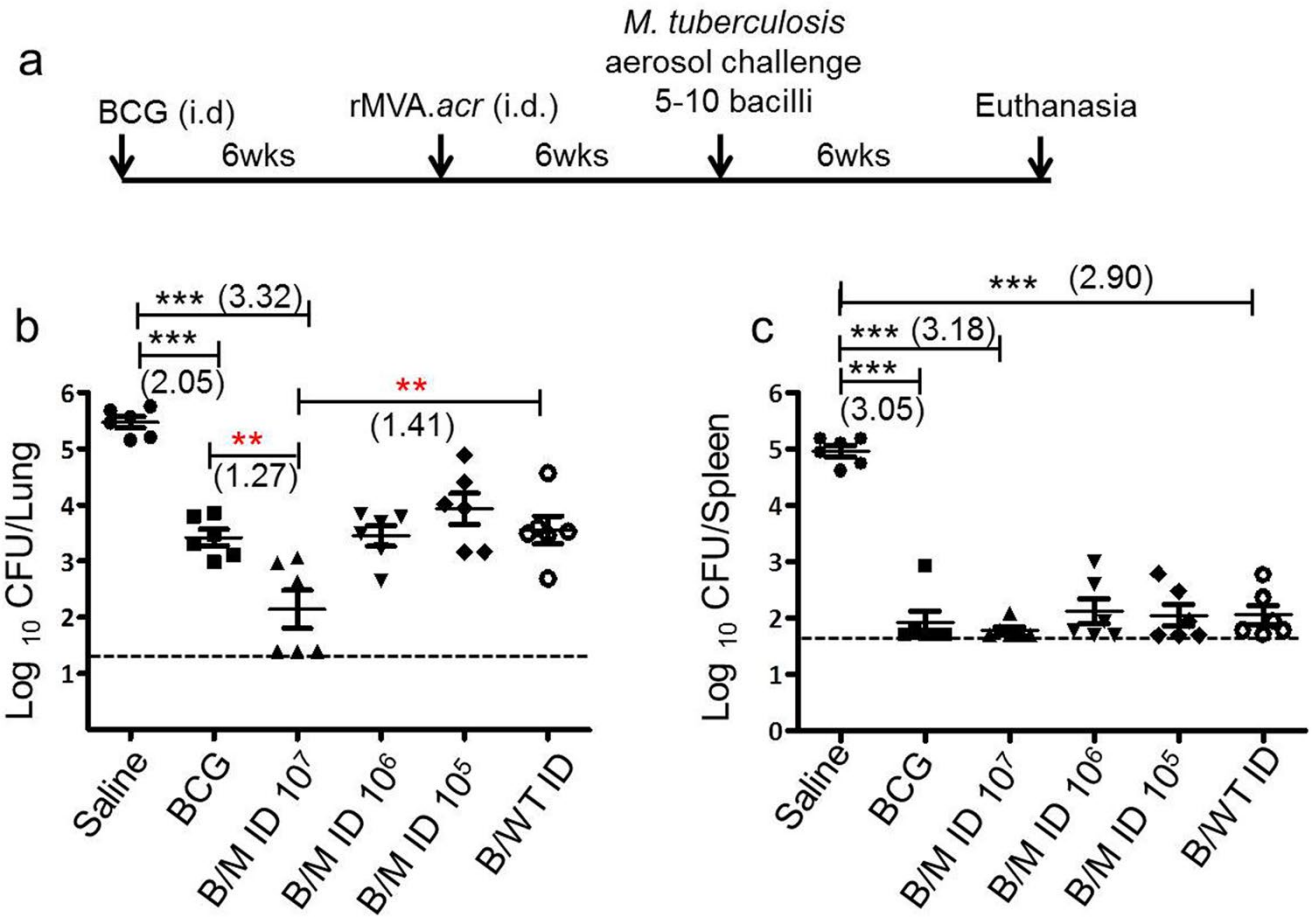
Evaluation of protective efficacy of rMVA.*acr* when administered intradermally against *M. tuberculosis* challenge in guinea pigs. (**a**) Experimental protocol employed to evaluate the protective efficacy of rMVA.*acr* as booster vaccine. The dot plot depicts the bacillary load in the lungs (**b**) and spleen (**c**) of vaccinated and saline (sham control) treated guinea pigs (n = 6) at 6 weeks post aerosol challenge. The bar depicts mean (± SE) for each group. Animals with undetectable bacilli were allotted a CFU value of 1.4 log_10_ (lung) and 1.7 log_10_ (spleen). The data was analysed by student’s unpaired *t -*test, ***p < 0.001, **p < 0.01 and *p < 0.05.

In order to assess the efficacy of rMVA.*acr* to boost BCG imparted protection, six weeks post BCG vaccination, guinea pigs were intradermally immunized with varying doses of rMVA.*acr* (1 × 10^5^ PFU, 1 × 10^6^ PFU and 1 × 10^7^ PFU) followed by aerosol challenge with *M. tuberculosis* H37Rv (Fig. 3a).

At 6 weeks post challenge, the sham immunized animals exhibited 5.47 log_10_ CFU in lungs. Immunization with BCG exhibited a significantly reduced CFU in lungs (2.05 log_10_ CFU reduction ***p < 0.001) in comparison to sham immunization. When BCG vaccinated animals were boosted intradermally with 1 × 10^7^ PFU of rMVA.*acr* (B/M ID 10^7^), it further reduced the pulmonary load (3.32 log_10_ fewer bacilli, ***p < 0.001) when compared with sham immunized animals demonstrating significantly enhanced protection. Moreover, the reduction in the bacillary load in the lungs of animals belonging to B/M ID 10^7^ regimen was noteworthy (1.27 log_10_ CFU fewer bacilli, **p < 0.01) in comparison to BCG vaccination (Fig. 3b). The effect of boosting as observed by the reduction in CFU was found to be antigen specific, since, boosting with wild type MVA (B/MVA.WT ID) did not show any enhancement of protection over BCG vaccination. Further, the remarkable protection afforded by boosting BCG vaccinated animals with rMVA.*acr* was observed to be dose dependent as boosting with 10-fold lower doses of rMVA.*acr* did not generate enhanced protection over BCG, although B/M ID 10^6^ and B/M ID 10^5^ regimens exhibited significant reduction in the lung bacillary load (2.02 log_10_ CFU and 1.54 log_10_ fewer bacilli, respectively), in comparison to the sham immunized animals (Fig. 3b).

In spleen, the sham immunized animals exhibited the highest bacillary load of 4.96 log_10_ CFU (Fig. 3c). The BCG vaccinated animals exhibited a significantly reduced bacillary load (1.91 log_10_ CFU) indicating a 3.05 log_10_ CFU reduction (***p < 0.001) as compared with sham immunized animals. When animals were immunized with B/M ID 10^7^ regimen, it exhibited 3.18 log_10_ reduction (***p < 0.001) in the bacillary load in comparison to sham immunized animals, thus, suggesting that boosting with rMVA.*acr* significantly limited the bacillary dissemination. Boosting BCG vaccinated animals with lower doses of rMVA.*acr*, also resulted in significant reduction in the splenic bacillary load in comparison to sham immunized animals, however, the extent of protection was comparable to animals immunized with BCG alone (Fig. 3c).

### Reduced pathological lesions observed on boosting BCG vaccinated guinea pigs with intradermal vaccination of rMVA.*acr*

**Figure 4:**
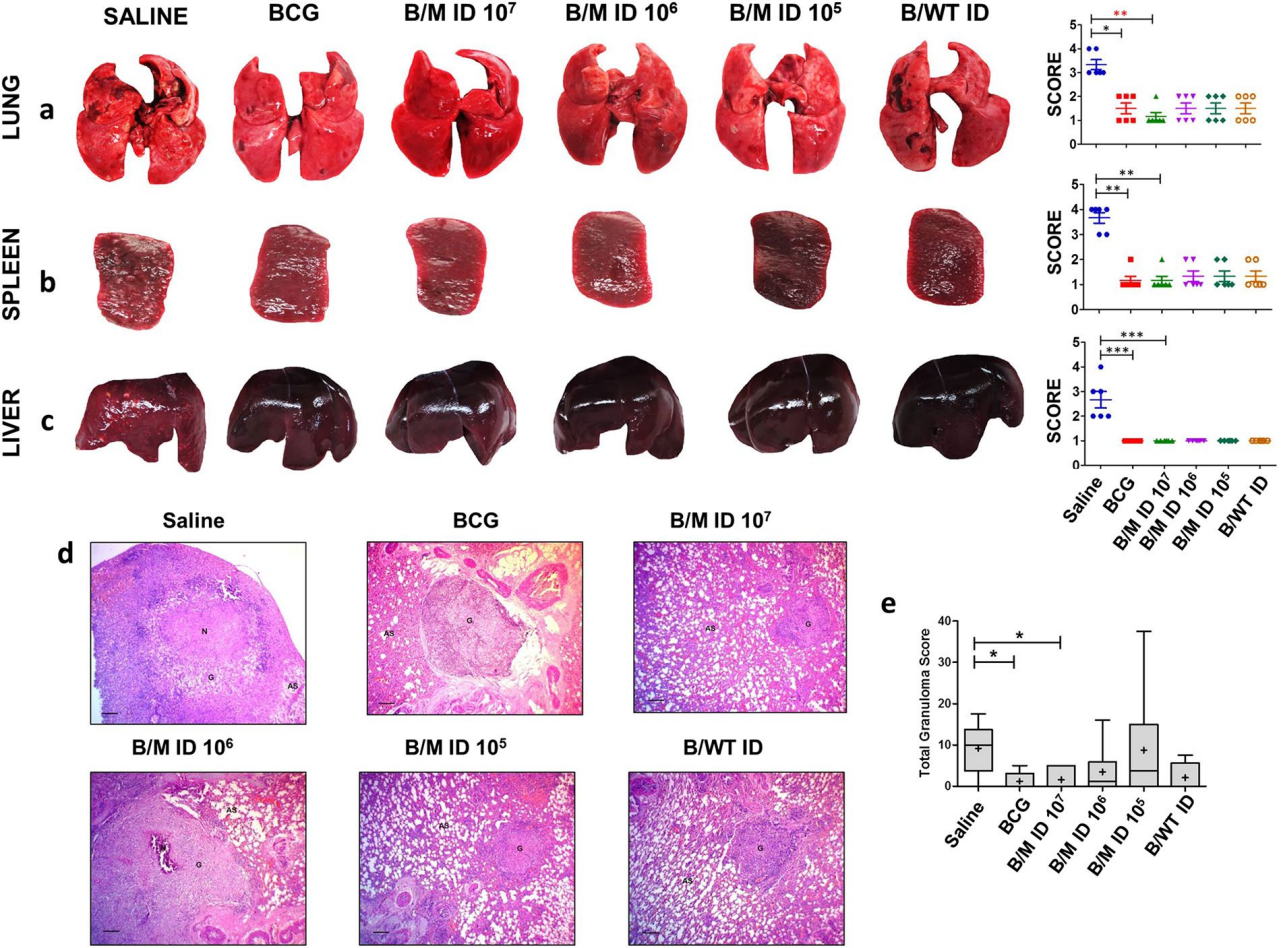
Gross and Histopathological analysis of various organs of guinea pigs at 6 weeks post infection. The figure depicts representative photographs and graphical depiction of gross scores of lung (**a**), spleen (**b**) and liver (**c**) of guinea pigs at 6 weeks post-infection. Each point represents score for an individual animal and the bar depicts median (±inter quartile range) for each group. For comparison of the gross pathological scores of various groups, the nonparametric Kruskal-Wallis test followed by Dunns comparison test was employed. ***p < 0.001 **p < 0.01 and *p < 0.05. (**d**) The figure depicts representative 40x magnification photomicrographs of formalin fixed, paraffin embedded and haematoxylin-eosin (H&E) stained 5 µm sections of lung tissue of vaccinated guinea pigs (n = 6) at 6 weeks post challenge. The scale bars depict 200 µm. AS and G denote alveolar spaces and granuloma, respectively. (**e**) The graphical representation of the total granuloma score of the lung sections is shown by box plot of 6 animals per group (the mean is represented by ‘+’, median value is denoted by horizontal line, box represents the inter quartile range and the minimum and maximum value is denoted by whiskers), *p < 0.05 and **p < 0.01 (Mann-Whitney test, two tailed).

On comparing the organs of the animals from different groups for gross pathological changes, the maximum damage was observed in sham immunized animals with numerous large tubercles and few areas of necrosis in the lungs (Fig. 4a). Intradermal vaccination with 1 × 10^7^ PFU of rMVA.*acr* to BCG immunized guinea pigs significantly reduced the pulmonary pathology, when compared to sham immunized as well as BCG vaccinated animals (Fig. 4a). Moreover, majority of the animals in B/M 10^7^ group showed negligible lesions in the lungs. Spleen from sham immunized animals also showed extensive involvement with numerous large tubercles, whereas, no visible lesions were observed in animals belonging to all the other vaccinated groups (Fig. 4b). On analysing the pathology in the liver, sham immunized animals showed numerous gross lesions, whereas animals belonging to all the vaccinated groups showed no pathological damage with no visible lesions (Fig. 4c).

Histopathological evaluation of lung tissues of animals belonging to various groups showed that sham immunized animals exhibited the maximum granuloma score with few necrotic granulomas predominantly formed of epitheloid cells (Fig. 4d and e). Vaccination of animals with BCG and other regimen significantly reduced the granulomatous lesions as observed by the reduction in the total granuloma score in comparison to saline treated animals (Fig. 4d and e). However, there was no difference in terms of histopathological damage and total granuloma score observed within all the vaccinated groups.

### Evaluation of protective efficacy of rMVA.*acr* when administered intramuscularly or intranasally to BCG vaccinated guinea pigs against *M. tuberculosis* challenge

**Figure 5:**
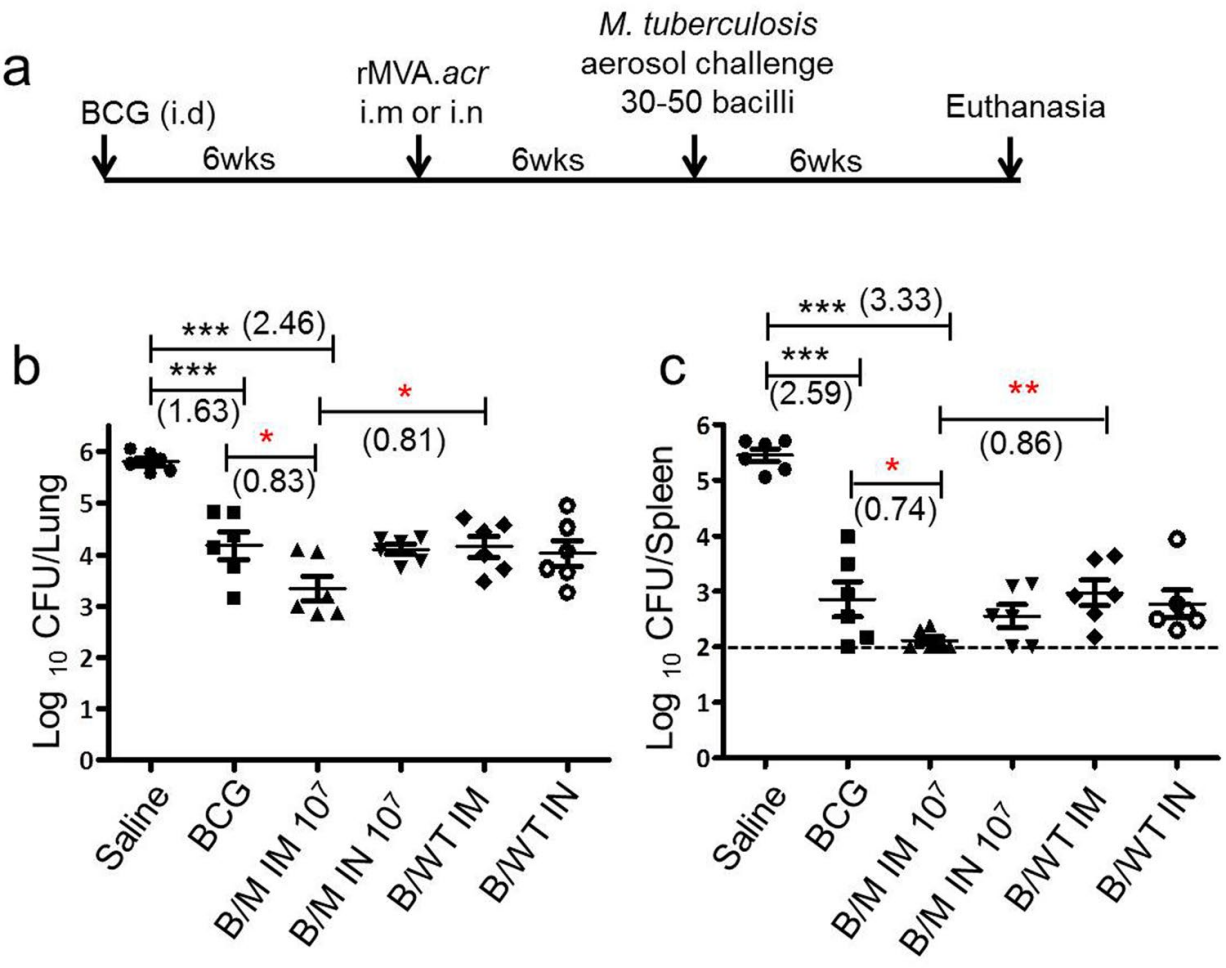
Assessment of bacillary load of BCG primed guinea pigs boosted with either intramuscular or intranasal delivery of rMVA.*acr*. (**a**) Experimental protocol employed to evaluate the protective efficacy of rMVA.*acr* as booster vaccine. The figure depicts the bacillary load in the lungs (**b**) and spleen (**c**) of vaccinated and saline (sham control) treated guinea pigs (n = 6) at 6 weeks post aerosol challenge. The bar depicts mean (± SE) for each group. Animals with undetectable bacilli were allotted a CFU value of 2.0 log_10_ (spleen). The data was analyzed by student’s unpaired *t*-test, ***p < 0.001, **p < 0.01 and *p < 0.05.

Intramuscular route of vaccination is one of the most common delivery methods employed for testing various vaccines^27,28^. Infact, most vaccines against other infectious diseases are delivered via the intramuscular route^27^. Besides, there is a renewed interest of delivering a vaccine via the needle free intranasal route. Administration of the vaccine via the intranasal route especially in context of tuberculosis is considered beneficial, since this route mimics the natural infection which might help in generating protective responses. Our results from the study involving intradermal administration of rMVA.*acr* demonstrated that a viral dose of 1 × 10^7^ PFU has an ability to impart superior protection than BCG alone against *M. tuberculosis* infection in guinea pigs. Hence, we evaluated the efficacy of rMVA.*acr* when given at a dose of 1 × 10^7^ PFU to boost the BCG imparted protection against *M. tuberculosis* when administered via intramuscular (i.m) and intranasal (i.n) route (Fig. 5a).

The sham immunized animals exhibited 5.80 log_10_ CFU in lungs. Immunization with BCG exhibited a significantly reduced bacillary load in the lungs by 1.63 log_10_ CFU (***p < 0.001) as compared to the sham immunized animals (Fig. 5b). Intramuscular boosting with 1 × 10^7^ PFU of rMVA.*acr* conferred enhanced protection by significantly reducing the pulmonary load by 2.46 log_10_ fewer bacilli, (***p < 0.001) when compared to sham immunized animals (Fig. 5b). Moreover, BCG vaccinated animals that received the rMVA.*acr* intramuscularly further exhibited significant reduction in the bacillary load by 0.83 log_10_ fewer bacilli, (*p < 0.05) in comparison to BCG vaccination (Fig. 5b). Importantly, the effect of boosting as observed by the reduction in the CFU was found to be antigen specific, since boosting with wild type MVA (B/MVA.WT IM) did not show any enhancement of protection over BCG vaccination. Further, when the α-crystallin based viral vaccine was administered by using intranasal route, it conferred superior protection when compared to sham immunized animals as observed by significantly reduced bacillary load (1.69 log_10_ fewer bacilli, ***p < 0.001), however, it did not confer any additional advantage over BCG vaccination (Fig. 5b).

In spleen, the sham immunized animals exhibited the highest bacterial burden of 5.45 log_10_ CFU (Fig. 5c). Immunization with BCG significantly reduced the bacillary load by 2.59 log_10_ CFU reduction (***p < 0.001) as compared to sham immunized animals. When animals were immunized with B/M IM 10^7^ regimen, it resulted in a 3.33 log_10_ CFU reduction (***p < 0.001), in comparison to the sham immunized animals, thus, suggesting that boosting with rMVA.*acr* via the intramuscular route significantly limits the bacillary dissemination. Moreover, the protection conferred by B/M IM 10^7^ regimen was noteworthy as it resulted in significantly reduced bacillary load (0.74 log_10_ fewer bacilli, *p < 0.05) when compared with BCG immunized animals (Fig. 5c). The influence of boosting with rMVA.*acr* was antigen specific as intramuscular administration of wild type MVA did not further reduce the splenic bacillary load in comparison to BCG vaccination. When BCG vaccinated animals were boosted with intranasal administration of rMVA.*acr*, the regimen markedly reduced the splenic bacillary load (2.89 log_10_ fewer bacilli, ***p < 0.001) as compared to sham immunized animals. The intranasal boosting of BCG vaccinated animals with rMVA.*acr* also resulted in 0.3 log_10_ fewer bacilli when compared to BCG vaccinated animals, however, the difference was not statistically significant.

### Pathological assessment of various organs upon booster vaccination with rMVA.*acr* via the intramuscular and intranasal route

**Figure 6:**
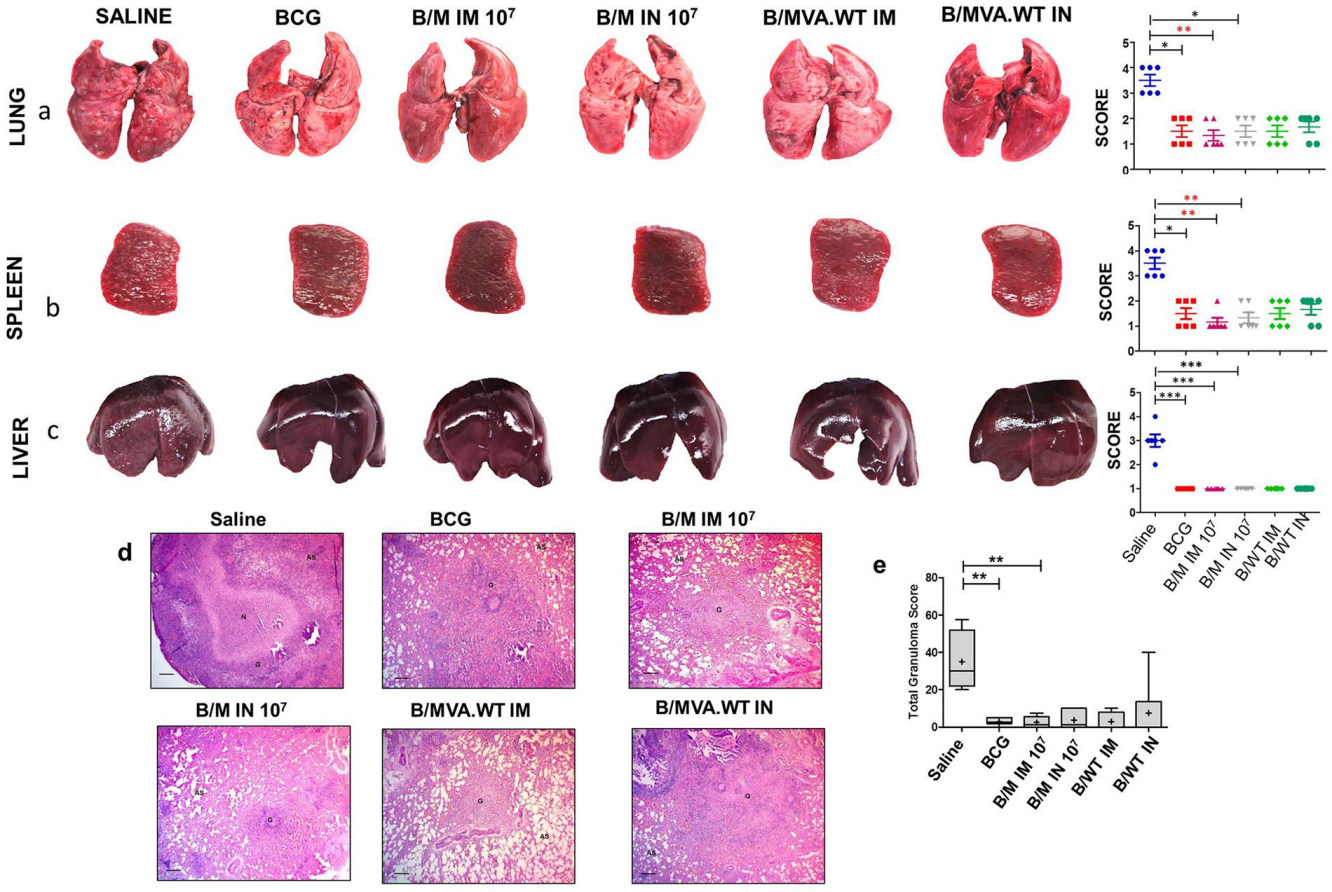
Evaluation of gross and histopathology of various organs of BCG vaccinated animals boosted with rMVA.*acr*. The figure depicts representative photographs and graphical depiction of gross scores of lung (**a**), spleen (**b**) and liver (**c**) of guinea pigs at 6 weeks post-infection. Each point in the graph represents score for an individual animal and the bar depicts median (± inter quartile range) for each group. For comparison of the gross pathological scores of various groups, the nonparametric Kruskal-Wallis test followed by Dunns comparison test was employed. ***p < 0.001 and **p < 0.01. (**d**) The figure depicts representative 40x magnification photomicrographs of formalin fixed, paraffin embedded and haematoxylin-eosin (H&E) stained 5 µm sections of lung tissue of vaccinated guinea pigs (n = 6) at 6 weeks post challenge. The scale bars depict 200 µm. AS and G denote alveolar spaces and granuloma, respectively. (**e**) The graphical representation of the total granuloma score of the lung sections is shown by box plot of 6 animals per group (the mean is represented by ‘+’, median value is denoted by horizontal line, box represents the inter quartile range and the minimum and maximum value is denoted by whiskers), *p < 0.05 and **p < 0.01 (Mann-Whitney test, two tailed).

Upon gross pathological examination, it was observed that the saline treated animals exhibited maximal damage with numerous large tubercules in the lungs along with few areas of necrosis (Fig. 6a). When the organs from the animals belonging to BCG, B/M IM 10^7^ and B/M IN 10^7^ groups were compared, it was observed that in comparison to the sham immunized animals the damage was markedly less, with minimal involvement and scanty tubercles. Moreover, B/M IM 10^7^ regimen exhibited minimal pulmonary involvement. Spleen from sham immunized animals showed extensive involvement with many large and small tubercles, whereas, lesions in the spleen of the animals belonging to B/M IM 10^7^ regimen were scanty and extremely small when compared to BCG, B/M IN 10^7^ and B/MVA.WT IM and B/MVA.WT IN immunized animals (Fig. 6b). On analysing the pathology in the liver, sham immunized animals showed numerous gross lesions (Fig. 6c). No significant difference in the gross pathological changes in the liver was observed between all the other vaccinated groups (Fig. 6c).

The extent of *M. tuberculosis* induced histopathological damage in the lungs was examined in each vaccine group at 6 weeks post infection. Lungs of saline treated animals showed maximum total granuloma score with a large number of necrotic granulomas majorly composed of epithelioid cells and lymphocytes (Fig. 6d and e). There was much reduced lung histopathology observed in animals belonging to other vaccination groups in comparison to sham immunized controls, however, there was no significant difference observed in the total granuloma score in animals belonging to BCG and other vaccination groups (Fig. 6d and e).

## Discussion

A perfect vaccine against tuberculosis would be the most cost effective intervention which can transform the TB control program throughout the world. Among the various approaches employed for the development of TB vaccines, heterologous prime boost approach which involves priming with BCG and boosting with any other vector has gained tremendous interest. Infact, the success of prime-boost approach is highlighted by the fact that most of the vaccine candidates that are currently being evaluated in clinical trials are based on this approach^29^.

We had previously developed a regimen involving BCG as primary vaccine boosted by a DNA vaccine expressing the latency associated antigen α-crystallin (B/D regimen)^13^. This regimen significantly reduced the bacillary load in lungs and spleen when compared to the unvaccinated animals^13^. Moreover, B/D regimen conferred a superior protection in comparison to the BCG vaccination in lung and spleen, at 10 weeks post-infection. In addition, B/D regimen continued to impart a superior protection over BCG even when the post-challenge period was prolonged to 16 weeks^13^. Besides, it also resulted in reduced antigen load and pathological damage. However, although DNA vaccines have shown very promising results in protection against tuberculosis in animal models, their performance in humans has not been as promising as in animal models. Hence, for taking the present work further, we developed a recombinant virus which is devoid of any selection marker which is also a prerequisite for any vaccine intended for human use.

To that note, the use of viral vectored candidate vaccines has been facilitated by the advances in the understanding of viral biology and the interaction of viruses with the host immune system. Recombinant poxviruses and adenoviruses expressing immunodominant antigens of *M. tuberculosis* have been evaluated for their ability to provide protection against TB^5,19,22,23,30,31^. In particular, MVA based vaccines have shown promising results against many infectious diseases including TB, HIV and malaria^32–35^. Recombinant MVA expressing *M. tuberculosis* antigen 85 A (MVA.85 A), has shown extremely encouraging results as a TB booster vaccine in various animal models against tuberculosis^19,22,23^.

In this study, we have developed and evaluated α–crystallin based prime boost vaccination strategy by employing MVA with an aim to improve the protection imparted by BCG against TB. The dose of a vaccine can play an important role in eliciting an optimal immune response which can influence the efficacy of a vaccine^36,37^. Besides, immunization route can also have a determining effect on vaccine efficacy. Recently, a lot of interest is developed among researchers to exploit the potential benefit of the intradermal route for vaccine delivery^38^. This is driven by the fact that the dermis or the epidermis layer of the skin consists of many antigen presenting cells (APCs), suggesting that vaccine delivery through this route will be more efficient in terms of eliciting a better immune response and concomitantly inducing better protection^38^. The dermal dendritic cells are the major group of immune cells that are responsible for antigen processing and presentation in the draining lymph nodes^38^. Uptake of the vaccine antigen by these dermal DCs induces many changes including increased expression of MHC molecules and co-stimulatory cells, secretion of various cytokines and increased ability to migrate to the draining lymph nodes^38^. All these factors provide impetus to favor the use of intradermal vaccination. Moreover, intradermal vaccination of MVA.85 A of BCG primed guinea pigs induced better protection against *M. tuberculosis* ^19^. Thus, in the first experiment, we have administered rMVA.*acr* intradermally at various vaccine doses ranging from 1 × 10^5^ PFU to 1 × 10^7^ PFU. Our results from this study demonstrated that intradermal delivery of rMVA.*acr* at a dose of 1 × 10^7^ PFU was able to efficiently boost the BCG induced protection in guinea pigs against *M. tuberculosis* infection. Our study demonstrated that the dose of vaccination indeed plays an important role as lower doses of rMVA.*acr* (1 × 10^5^ PFU and 1 × 10^6^ PFU) were unable to generate any enhancement of protection over BCG vaccination (Fig. 3). Our results showed that BCG vaccination provided excellent protection against dissemination to the spleen, however, boosting of BCG with rMVA.*acr* did not confer any additional improvement in the efficacy of BCG irrespective of the viral dose. A probable reason for this could be the low dose (5–10 bacilli) of challenge that was employed in this study against which BCG demonstrates sterilizing effect in the spleen. Hence, assessment of efficacy of any booster vaccine over BCG would be more discriminative in a system that provides a suitable window to evaluate any improvement in protection imparted by BCG alone. Evaluating booster vaccines to BCG against higher challenge dose should provide a margin for improvement.

Thus, in our next study, we employed an increased dose of challenge from 5–10 bacilli to 30–50 bacilli. Besides, we also compared two different routes of vaccination (intramuscular and intranasal route) of rMVA.*acr* administered at 1 × 10^7^ PFU. Our results demonstrated that intramuscular administration of rMVA.*acr* to BCG primed guinea pigs (B/M IM 10^7^ regimen) exhibited superior protection in comparison to BCG alone (Fig. 5). However, intranasal vaccination of rMVA.*acr* did not enhance the protection generated by BCG in guinea pigs.

In conclusion, this preliminary study demonstrated the potential use of rMVA.*acr* as a TB booster vaccine. Administration of rMVA.*acr* either via the intradermal mode or intramuscular route augments the BCG imparted protection against *M. tuberculosis* in guinea pigs. Further, evaluation of this vaccination strategy for survival assays would be necessary to understand the implication of this regimen in providing long term protection in guinea pigs. In addition, studies to discern the immune mechanisms underlying this protection would help in identifying biomarkers as well as would aid in the development of efficient booster vaccines for BCG vaccinated individuals.

## Methods

### Virus and plasmid

MVA virus stock (MVA 1974/ NIH-Clone 1) was kindly provided by Prof. Bernard Moss at the National Institutes of Allergy and Infectious Diseases (NIAID), the National Institutes of Health (NIH), Bethesda, USA. pSC65 was provided by Prof. Sekhar Chakrabarty, National Institute of Cholera and Enteric Diseases (NICED), Kolkata, India. pLAS2 was a generous gift from Indian Immunologicals Limited, Hyderabad, India.

### Site directed mutagenesis

The primers used for the generation of *Spe*I restriction site are listed in Table 1. Polymerase chain reaction (PCR) amplification of pSC65.*gfp* was carried out by using the primers designed for mutagenesis followed by *Dpn*I digestion followed by the transformation of the reaction mixture in *E. coli* XL-1Blue cells. The mutations were confirmed by DNA sequencing and restriction digestion.

### Primary CEF cell line

The CEF cell monolayers were prepared according to the standard protocol^25^. Specific pathogen free (SPF) eggs were purchased from Venky’s Limited, Pune, India for preparing primary CEF cell lines. Briefly, 9–11 eggs were incubated at 37 °C for 10–11 days before further use. The shells were wiped with 70% ethanol, pierced with sterile needle and the hole was enlarged in a circular pattern with the help of sterile scissors until an intact circle of shell could be lifted from the egg. The chorioallantoic membrane was removed from each egg and the embryos were placed in a 90 mm sterile petri dish. The head and limbs were removed and the rest of the body was transferred into another 90 mm sterile petri dish containing 10 ml Modified Eagle’s Medium -1X GlutaMAX (Invitrogen life technologies, CA, USA) supplemented with 1% antibiotic-antimycotic mix (ABAM). Further, the embryos were minced through a 20 ml syringe (without an attached needle) into an autoclaved 500 ml reagent bottle followed by trypsinization (12.5 ml of trypsin (TrypLE Select CTS, Invitrogen Bioservices, India Pvt. Ltd.) diluted in 25 ml of DPBS; pre-warmed at 37 °C). Following incubation for 40 min at room temperature along with constant stirring, the suspension was filtered through cheese cloth into a 50 ml falcon tube and spun at 2500 rpm for 10 min at room temperature. The cell pellet so obtained was washed in 50 ml of modified eagle’s medium (MEM) supplemented with 10% gamma-irradiated fetal bovine serum (FBS, Morgate Biotech, Australia) and 1% ABAM (MEM-10) and was pelleted by centrifuging at 2500 rpm for 10 min at RT. Subsequently, the cell pellet was resuspended in 20 ml MEM-10 by tituration. 2 ml of this cell suspension mixed in 10 ml of MEM-10 was added per T-75 tissue culture flask, and incubated at 37 °C, 5% CO_2_. Next day, media was discarded from each flask to remove non-adherent cells & fresh MEM-10 was added. The cell line was further incubated at 37 °C, 5% CO_2_ until 90–100% confluency was attained. The fibroblast cells thus obtained were passaged once before use.

### Cloning of *gfp* gene, α-crystallin gene and direct repeat sequence in pSC65

*lac*Z gene was excised out by using *Xho*I and *Bam*HI restriction enzymes to generate pSC65.Δ*lacZ* (Supplementary Fig. S1). *gfp* gene was amplified from pLAS2 plasmid by using 5′atcgctcgagatggtgagcaagggcgaggag 3′ containing *Xho*I site as the forward primer and 5′atcgggatccataaaaatcatcacttgtacagctcgtccatgc 3′containing *Bam*HI site as the reverse primer. *Bgl*II enzyme was used to excise out the α-crystallin gene from pLit38.acr^12^. The excised fragment was treated with DNA polymerase I, large (Klenow) fragment to generate blunt ends and ligated at *Sma*I site in pSC65.*gfp*.SpeI (Supplementary Fig. S3b). *Msc*I and *Pac*I digestion was carried out to confirm both, the cloning as well as orientation of α-crystallin gene. 5′ ggatacactagtttctgtcagcgtatggc 3′ containing the *Spe*I site as forward primer and 5′ gatcactagtgagtcgatgtaacac 3′ containing the *Spe*I site as reverse primer were used to amplify 260 bp (direct repeat) of the flank II (TKR) region (Table 1). The amplified product was then cloned at *Spe*I site in the plasmid pSC65.*gfp*.*acr* to generate pSC65.*gfp*.*acr*.DR (Supplementary Fig. S5).

### Generation of rMVA.*gfp.acr*

MVA-WT was propagated on primary CEF cell line. 2 μg plasmid DNA (pSC65.*gfp.acr*.DR) was pre-complexed with 4 μg Lipofectamine 2000 reagent in 200 μl serum-free MEM for 20 min at RT. This pre-complexed mix was added to CEF cell lines infected with MVA-WT at a MOI of 0.1. The cells were left for incubation at 37 °C for 24 hours in a CO_2_ incubator to allow the recombination event to take place (Fig. 1b). The transfected cells were harvested by scraping the cells and subjected to three cycles of freeze-thaw. Various 10 fold dilution of the transfected mix was incubated on fresh CEF cell line for 6 hours and subsequently overlaid with 1% methyl cellulose to restrict the growth of the virus for 48 hours. Various recombinant virus foci expressing GFP, as observed under the fluorescence microscope, were picked by using sterile toothpick. DNA was isolated from these foci by using Qiagen DNA isolation micro kit as per manufacturer’s instructions and PCR was carried out to confirm the identity of the clone by using primers (GFP-F and TKL-R) designed to selectively amplify the region from *gfp* gene to TKL flank (Fig. 1d, Table 1).

The recombinant virus obtained above is not a pure population and may contain both the parental and recombinant virus. Hence, it was necessary to plaque purify the recombinant virus by serially passaging it on CEF cell line in order to dilute out the wild type virus and clonally purify the recombinant MVA virus. Hence, the recombinant virus obtained above (Fig. 1d), was passaged 7 times on 1° CEF cell line. At each passage, PCR analysis was carried out to characterize the recombinant clone for the presence of α-crystallin gene and to confirm site-specific recombination. The integrity of the final clone named rMVA.*gfp.acr* was confirmed via PCR analysis by using various sets of primers as listed in Table 1.

### Limiting dilution method

Marker-free recombinant virus was isolated by using the limiting dilution method^39^. For this, the rMVA.*gfp.acr* was amplified on confluent CEF cell lines several times and the crude viral stock was prepared by harvesting these cells. It is expected that *gfp* will be removed (on several successive rounds of amplification) by intragenomic recombination of direct repeats generating two kinds of virus isolates: (i) Recombinant virus containing the entire gene cassette (rMVA.*gfp.acr*) and (ii) Recombinant virus from which the marker gene (*gfp*) is excised due to recombination between direct repeat sequences (rMVA.*acr*) (Fig. 1f). The titre of rMVA.*gfp.acr* was determined by immunostaining. To isolate the rMVA which has lost selection marker *gfp* (rMVA.*acr*), an average of 1 or 0.5 virus particle was distributed into individual wells of 96-well plates coated with a monolayer of CEF cell line. After 48 hours, nine wells showed visible cytopathy but no GFP expression. The DNA from the cells in these nine wells were collected and further screened by PCR. The final clone (rMVA.*acr*) was characterized by fluorescence microscopy, PCR analysis (primers described in Table 1) and immunoblotting.

### Immunostaining

MVA does not form distinct plaques^26^, therefore, to determine the virus titre of the MVA-WT and recombinant MVA, immunostaining was performed by using antibodies against vaccinia virus and α-crystallin, respectively. Ten-fold serial dilutions of freeze-thawed viral suspension were made in 1 ml of MEM supplemented with 1% FBS and 1% ABAM (MEM-1). 90–100% confluent CEF cell monolayers were infected with 1 ml of diluted virus suspension per well for 6 hours at 37 °C in a CO_2_ incubator, following which 2 ml of 1% methylcellulose (reconstituted in MEM-1) was added and left for further incubation at 37 °C for 72 hours in a CO_2_ incubator. Subsequently, the cells were washed with 1X PBS and fixed with a mixture of acetone & methanol (1:1) for 2 min. After fixing, 2 ml of blocking buffer (2% FBS in 1X PBS) was added and incubated for 30 min at room temperature with gentle rocking. Following this, the cells were stained with primary antibodies, rabbit anti-Vaccinia Virus polyclonal antibody at a dilution of 1:2000 (AbD Serotec, A Bio-Rad company) to determine the plaque forming units or rabbit anti-α-crystallin polyclonal antibody at a dilution of 1:250, (prepared in our laboratory) to evaluate for the expression of α-crystallin, for 3 hours at room temperature with gentle rocking. Primary antibody was removed and the cells were washed with PBS for three times. Subsequently, 1 ml of horse raddish peroxidase conjugated secondary antibody at a dilution of 1:2000 in PBS was added and incubated for 30–45 min at room temperature with gentle rocking. Cells were then washed with PBS for three times. Antibody bound antigens were then detected with DAB (1 mg/ml in PBS containing 0.3% H_2_O_2_) as a chromogenic substrate for HRP.

### Immunoblotting

To analyse the expression profile of the target antigen, α-crystallin, CEF cell monolayers were infected with rMVA.*acr* at a MOI of 10. The cells were harvested at 48 hours post infection and analysed by immunoblotting. Briefly, the cells were dislodged from the surface of the tissue culture plate by scraping and subjected to centrifugation at 2500 rpm for 5 min. The cell pellet was resuspended in 200 μl of lysis buffer (1% Nonidet P-40, 0.1% SDS, 50 mM Tris-HCl pH 8.0, 150 mM NaCl 0.5% sodium deoxycholate containing 1.5X protease inhibitor). Estimation of protein was carried out by Bradford’s method^40^. 50 μg of protein lysate was electrophoresed on 15% SDS-polyacrylamide gel and subjected to immunoblotting by using anti-α-crystallin mouse monoclonal antisera (Biodefense and Emerging Infections Research Resources Repository, BEI Resources, USA).

### Genetic stability studies

Nine individual isolates from low passage stock of rMVA.*acr* were serially passaged individually in CEF cell lines eight times. Briefly, confluent CEF cell line was infected with the virus at a MOI of 1 PFU/cell, grown for 72 hours. The cells were harvested and again used for infecting fresh CEF cell line with a MOI of 1 PFU/cell. This procedure was repeated sequentially for eight subsequent passages. The genetic stability as measured by the yields of virus was assessed by using PCR analysis and immunostaining.

### Bacterial strains and preparation of vaccines

*E. coli* XL-1B cells were grown in Luria Bertani (LB) broth at 37 °C with constant shaking at 200 rpm. Ampicillin (50 µg/ml) was added to culture media when required. *M. bovis* BCG (Danish strain, BCG laboratories, Chennai, India) and *M. tuberculosis* (H37Rv strain, ATCC no. 25618, AIIMS, New Delhi, India) were grown to mid-log phase in Middle Brook (MB) 7H9 media supplemented with 0.5% glycerol, 1X ADC (albumin-dextrose-catalase, BD Difco) and 0.05% Tween 80, followed by the preparation of PBS stocks which were stored at −80 °C, until further use. For the enumeration of stock CFU, appropriate dilutions were plated in duplicates on MB7H11 agar supplemented with 1X OADC (oleic acid-albumin-dextrose-catalase, BD Difco) and 0.5% glycerol.

rMVA.*acr* was prepared according to standard protocol^25,26^. For preparing the high titre stocks of rMVA.*acr*, 90- 100% confluent CEF cell line in 25 T-175 tissue culture flasks were infected with MVA virus at a MOI of 0.1. After 72 hours, visible cytopathy was observed and the cells were scraped and transferred to 50 ml falcons. After centrifugation at 2500 rpm for 10 min, the pellet (crude virus lysate) was resuspended in MEM-1 and stored as aliquots at −80 °C. Subsequently, the pellet was subjected to three rounds of freeze-thaw cycle. The crude viral lysate generated from rMVA.*acr* was transferred to a tight-fitted dounce homogenizer. The suspension was dounced five times on ice (5 strokes) and the procedure was repeated for a total of five sets. The suspension was allowed to cool in between sets of strokes. Then, the virus suspension was centrifuged for 10 min at 2500 rpm at 4 °C. The supernatant so obtained was further subjected to 36% sucrose cushioning by filling half a volume of centrifuge tube with 36% sucrose and overlaying with equal volume of virus supernatant. This was centrifuged for 2 hours at 16,000 rpm at 4 °C. The supernatant containing sucrose and cell debris was discarded and the pellet was resuspended in 1X PBS. To determine the viral titre of the rMVA.*acr*, immunostaining was carried out by using polyclonal antibodies against α-crystallin and Vaccinia virus as described in the above section.

### Animals

Female guinea pigs (Dunkin Hartley strain, ~200–300 g) were procured from Disease Free Small Animal House, Lala Lajpat Rai University, Hissar, India. Animals were maintained and housed in individually ventilated cages under standard conditions in the Bio safety laboratory III facility at University of Delhi South Campus and were provided with food and water *ad libitum*. All animals were allowed to acclimate and allotted randomly in different experimental groups before initiating the experiment.

### Ethics Statement

All the experimental protocols included in this study were reviewed and approved by the Institutional Animal Ethics Committee of the University of Delhi South Campus (Ref No. 1/IAEC/AKT/Biochem/UDSC/ 7.8.2013 and Ref No. 25/IAEC/AKT/Biochem/UDSC/ 17.8.2015). The animals were routinely cared for, according to the guidelines of Committee for the Purpose of Control and Supervision of Experiments on Animals (CPCSEA), Ministry of Environment, Forests and Climate change, Government of India. Attempts were made to reduce the pain and suffering of the animals employed in this study. Specific pathogen free eggs were purchased from the vendor, Venky’s India Limited (SPF egg division), Pune, India. The age of the chick embryo eggs at the time of purchase was zero day which were allowed to incubate at 37 ^o^C with intermittent turning of the platform every 30 minutes for a period of 10 days before use. CEF cell line was prepared by using 10^th^ day eggs. According to the National Institutes of Health, USA, Office for Protection from Research Risks (OPRR), it is stated that “Public Health Service (PHS) Policy is applicable to proposed activities that involve live vertebrate animals. While embryonal stages of avian species develop vertebrae at a stage in their development prior to hatching, OPRR has interpreted “live vertebrate animal” to apply to avians (e.g., chick embryos) only after hatching”^41^. Hence, ethical approval for the use of chick embryo eggs for the preparation of CEF cell line from day 10 eggs was not required. For intradermal (i.d) and intramuscular immunization (i.m), animals were injected with various vaccines in 100 µl of saline. For intranasal delivery (i.n), animals were lightly anesthetized i.m with a mixture of ketamine and xylazine (7.5 mg/kg and 0.625 mg/kg, respectively)^30^. For i.n delivery, 25 µl of diluted virus vaccine was administered to each nare for inhalational inoculation. Animals were euthanized by using CO_2_ asphyxiation whenever required during the daytime in Bio safety laboratory III. No animals died prior to the experimental timepoint.

### Immunization and Infection

Two sets of experiments were carried out. In the first experiment, groups of 6 guinea pigs were immunized with one of the following: (i) 100 µl of saline by intradermal (i.d.) route (control group), (ii) 5 × 10^5^ CFU of BCG in 100 µl of saline by i.d. route, (iii) 5 × 10^5^ CFU of BCG once, followed by a booster dose of either 1 × 10^7^ PFU or 1 × 10^6^ PFU or 1 × 10^5^ PFU rMVA.*acr* in 100 µl of saline by using i.d. route at 6 week interval (B/M ID regimen) and (iv) 5 × 10^5^ CFU of BCG once, followed by a booster dose of MVA.WT (1 × 10^8^ PFU) in 100 µl of saline by using i.d. route at 6 week interval (B/MVA.WT ID). Guinea pigs were challenged at 6 weeks after the last immunization with 5–10 bacilli of virulent *M. tuberculosis* H37Rv via the respiratory route in an aerosol chamber (Inhalation exposure system A4224, Glas-Col Inc, USA).

In the second set of experiment, groups of 6 guinea pigs were immunized with one of the following: (i) 100 µl of saline by intradermal (i.d.) route (control group), (ii) 5 × 10^5^ CFU of BCG in 100 µl of saline by i.d. route, (iii) 5 × 10^5^ CFU of BCG once, followed by a booster dose of rMVA.*acr* (1 × 10^7^ PFU) by using i.m. route at 6 week interval (B/M IM 10^7^), (iv) 5 × 10^5^ CFU of BCG once, followed by a booster dose of rMVA.*acr* (1 × 10^7^ PFU) by using i.n. route at 6 week interval (B/M IN 10^7^), (v) 5 × 10^5^ CFU of BCG once, followed by a booster dose of MVA.WT (1 × 10^8^ PFU) by using i.m. route at 6 week interval (B/MVA.WT IM) and (vi) 5 × 10^5^ CFU of BCG once, followed by a booster dose of MVA.WT (1 × 10^8^ PFU) by using i.n. route at 6 week interval (B/MVA.WT IN). Guinea pigs were challenged at 6 weeks after the last immunization with 30–50 bacilli of virulent *M. tuberculosis* H37Rv via the respiratory route in an aerosol chamber (Inhalation exposure system A4224, Glas-Col Inc, USA).

### Bacterial CFU enumeration

Guinea pigs were euthanized by CO_2_ asphyxiation at 6 weeks post challenge. The lung and spleen were aseptically removed. Left caudal lung lobe and whole spleen were processed for enumeration of the bacillary load. Briefly, tissues were homogenized in 5 ml of normal saline buffer and serial dilutions of the homogenates were plated on to MB7H11 agar plates in duplicates and incubated at 37 °C in a CO_2_ incubator for 3–4 weeks. The number of colonies was counted and expressed as log_10_ CFU/organ.

### Pathological evaluation

After aseptically dissecting the animals, the lung, liver and spleen were examined for gross pathological damage by using modified Mitchison scoring system (1–4 grading system) on the basis of tissue involvement, areas of inflammation, number and size of tubercles and the extent of necrosis^42^. The lung lobes were fixed in 10% buffered formalin and subsequently embedded in paraffin wax. Sections of 5 μm thickness were cut on to glass slides and stained with Haematoxylin and Eosin for histo-pathological examination by a certified pathologist (who was blinded about the identity of the various experimental group) according to the criteria described previously by Lasco *et al*.^43^. Briefly, a score of 5 was assigned to the granuloma with necrosis, granuloma without necrosis was given a score of 2.5 and granuloma with fibrous tissue was given a score of 1. All granulomas in each section were scored and the scores were added up to obtain a total granuloma score of lung of each animal.

### Statistical analysis

For comparison between the groups for the determination of bacillary load in the lungs or spleen of experimental guinea pigs, unpaired t-test (two-tailed) was employed. For comparison between the groups for the analyses of total granuloma score, Mann-Whitney test (two-tailed) was employed. For the analysis of the gross pathological damage between the groups, nonparametric Kruskal-Wallis test followed by the Dunn’s multiple comparison test was employed. Differences were considered significant, when *p* < 0.05. For statistical analysis and generation of graphs, GraphPad Prism 5 software (Version 5.01; GraphPad Software Inc., CA, USA) was used.

## Electronic supplementary material

Supplementary Information
