# Supplementary material for: Boosting with recombinant MVA expressing M. tuberculosis α-crystallin antigen augments the protection imparted by BCG against tuberculosis in guinea pigs
Source: Sci Rep. 2017 Dec 11;7:17286. doi: 10.1038/s41598-017-17587-5 (PMC5725557; doi:10.1038/s41598-017-17587-5)
Supplement: Supplementary file 1 — Supplementary Information [file 41598_2017_17587_MOESM1_ESM.pdf]

Boosting with recombinant MVA expressing α-crystallin antigen of *M. tuberculosis* augments the protection imparted by BCG against tuberculosis in guinea pigs.

Prachi Nangpal[1], Ritika Kar Bahal[1] and Anil K. Tyagi[*1,2]

[1]Department of Biochemistry, University of Delhi South Campus, Benito Juarez Road, New Delhi, India

[2]Vice Chancellor, Guru Gobind Singh Indraprastha University, Sector 16-C, Dwarka New Delhi, India

***To whom correspondence may be addressed: Prof. Anil K. Tyagi**

Department of Biochemistry, University of Delhi South Campus, Benito Juarez Road, New Delhi-110021, India. Tel.: 91-11-24110970; Fax: 91-11-24115270; E-mail: aniltyagi@south.du.ac.in

Supporting information

Supplementary Figure S1

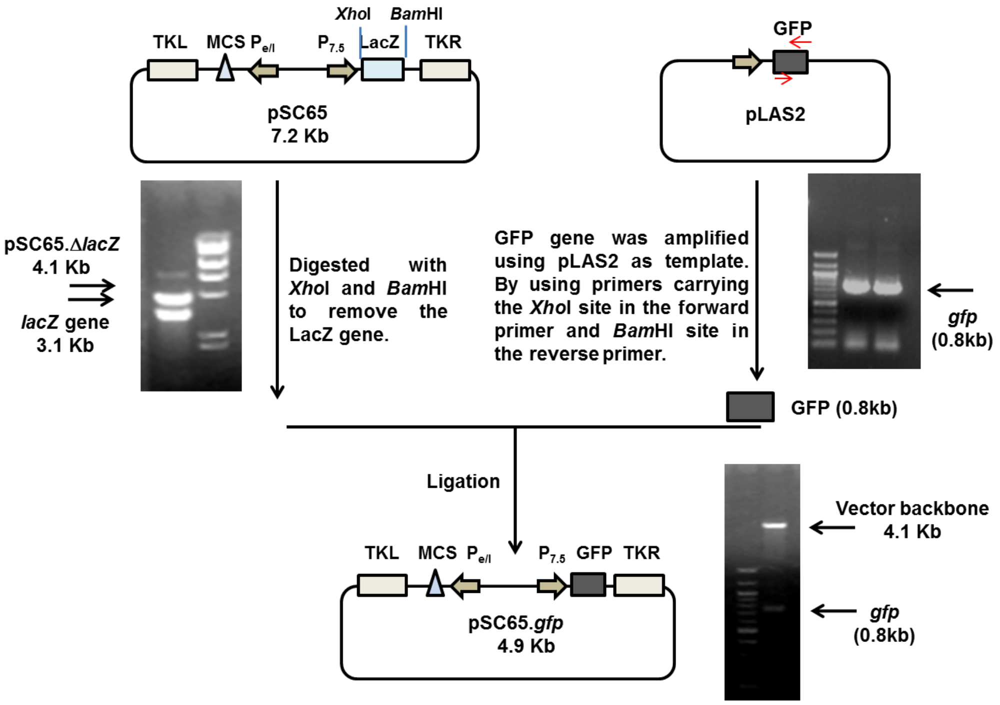

Figure S1: Generation of pSC65.*gfp* from the vector pSC65. *lacZ* gene was excised out by restriction digestion with *Xho*I and *Bam*HI and replaced with *gfp* gene (obtained from pLAS-2) to generate pSC65.*gfp*. The cropped gels are used in the figure, and full-length gels are presented in Supplementary Fig. S2.

Supplementary Figure S2

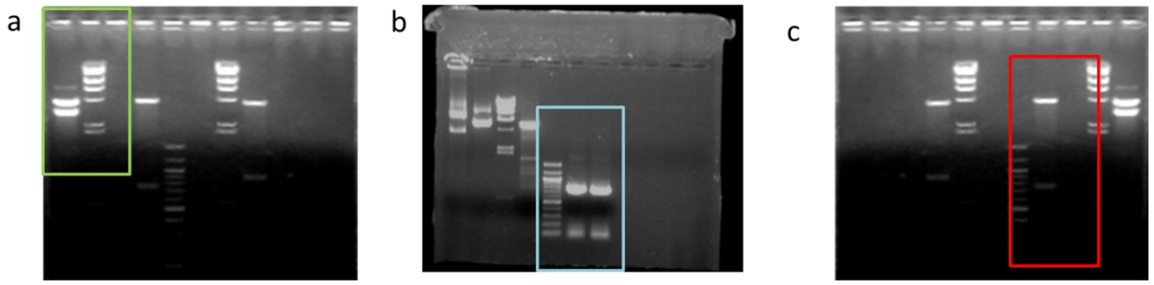

Figure S2: Full length gels of Supplementary Figure S1. a. The green highlighted portion of the gel show the cropping location which represents the lacZ gene excision. b. The blue highlighted portion of the gel show the cropping location which represents the amplification of *gfp* gene. c. The red highlighted portion of the gel show the cropping location which represents cloning of the *gfp* gene. Brightness has been adjusted during the processing of the gel.

Supplementary Figure S3

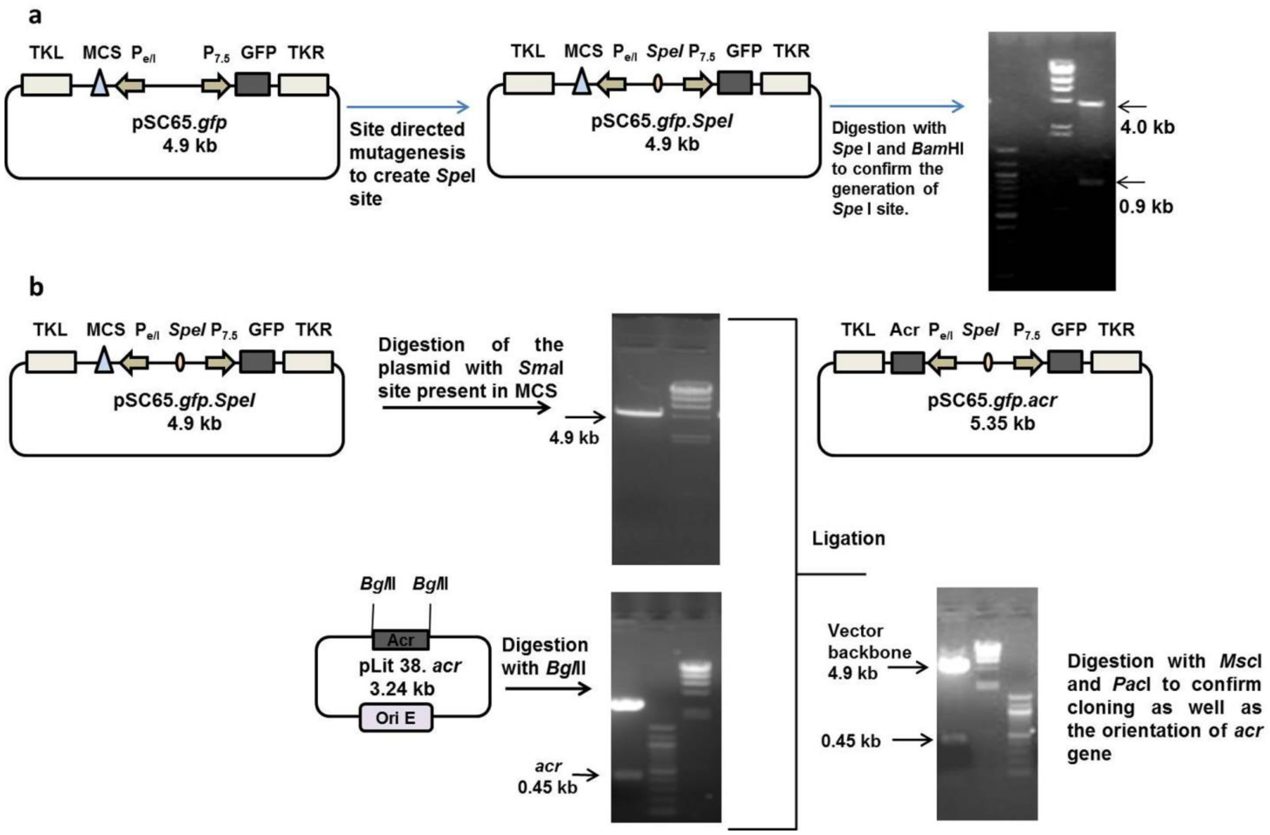

Figure S3: Generation of pSC65.*gfp.acr* (a) Site directed mutagenesis was carried out to create a *SpeI* restriction site in pSC65.*gfp* in order to clone a direct repeat sequence. (b) The 0.45 kb α-crystallin gene was digested out of the vector pLit.38.*acr* by using *Bgl*II and subcloned at *Sma*I site into pSC65.*gfp.Spe*I to generate pSC65.*gfp.acr*. The cropped gels are used in the figure, and full-length gels are presented in Supplementary Fig. S4.

Supplementary Figure S4

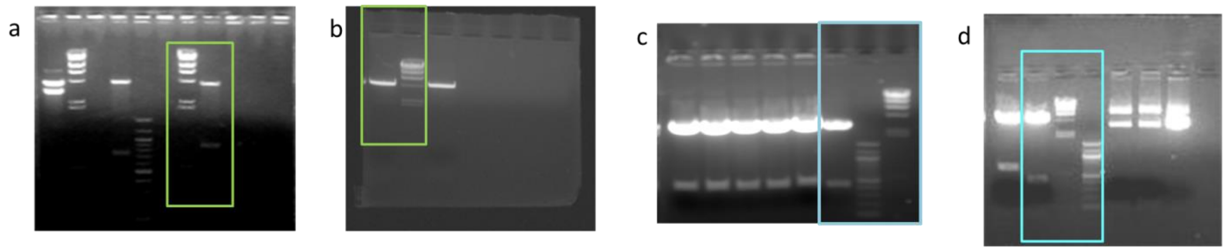

Figure S4: Full length gels of Supplementary Figure S3. a. The green highlighted portion of the gel show the cropping location which represents the generation of *SpeI* site. b. The green highlighted portion of the gel show the cropping location which represents the *Sma*I digestion of the plasmid. b. The blue highlighted portion of the gel show the cropping location which represents the excision of *acr* gene from the pLit38.acr plasmid. c. The blue highlighted portion of the gel show the cropping location which represents cloning of the *acr* gene. Brightness has been adjusted during the processing of the gel.

Supplementary Figure S5

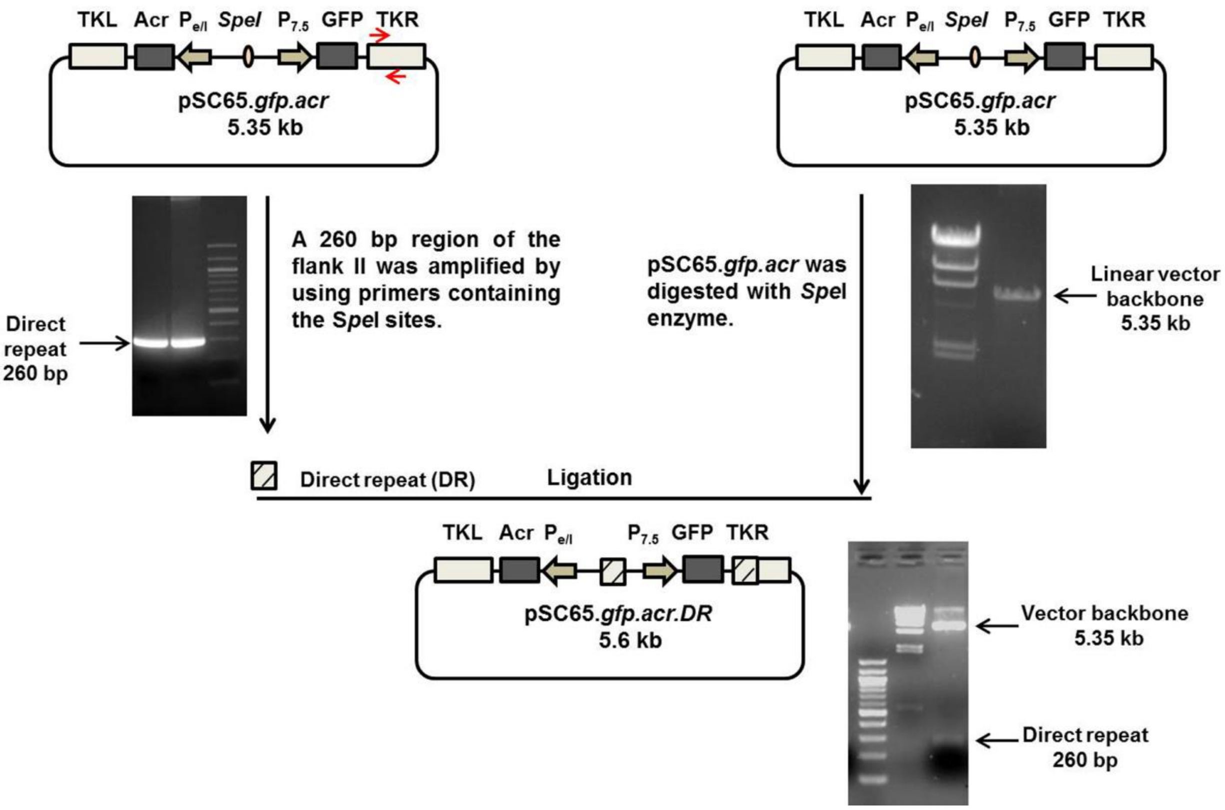

Figure S5: Cloning of direct repeat sequence in pSC65.*gfp*.acr to generate the plasmid transfer vector pSC65.*gfp.acr*.DR. A 260 bp segment of the thymidine kinase (TK) right region was amplified by using primers containing *Spe*I sites. The amplified product was cloned in pSC65.*gfp*.*acr* to generate pSC65.*gfp*.*acr*.DR. The cropped gels are used in the figure, and full-length gels are presented in Supplementary Fig. S6.

Supplementary Figure S6

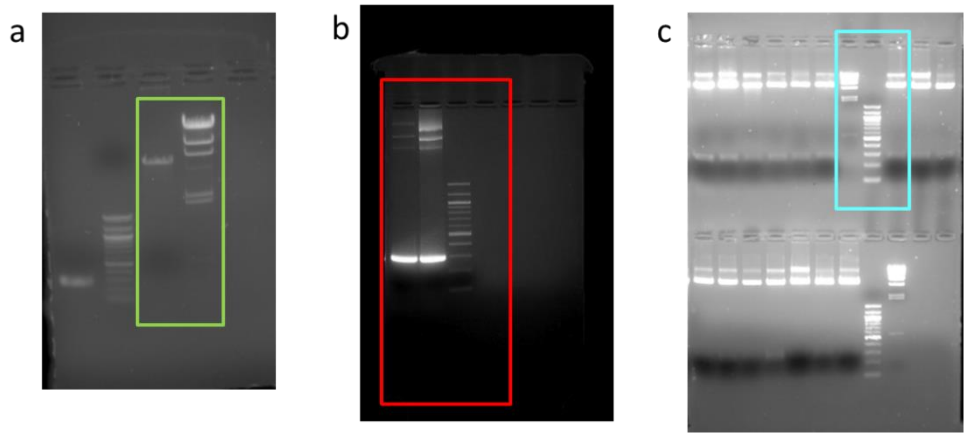

Figure S6: Full length gels of Supplementary Figure S5. a. The green highlighted portion of the gel show the cropping location represents the digestion by *Spe*I enzyme. b. The red highlighted portion of the gel show the cropping location which represents amplification of direct repeat sequence. c. The cyan highlighted portion of the gel show the cropping location which represents cloning of the direct repeat sequence. Brightness has been adjusted during the processing of the gel.

Supplementary Figure S7

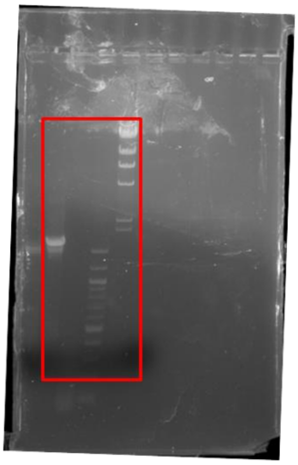

Figure S7. Full length gels of Figure 1d. The red line shows the cropped location of the gel which is employed in the manuscript. Brightness has been adjusted during the processing of the gel picture. Brightness has been adjusted during the processing of the gel.

Supplementary Figure S8

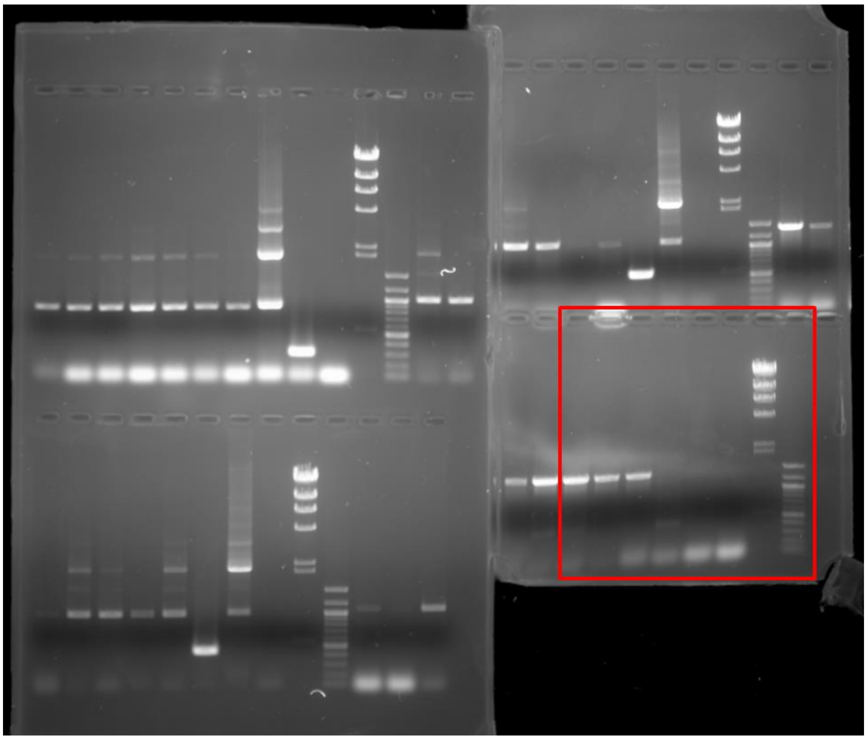

Figure S8. Full length gels of Figure 1e (i). The red line shows the cropped location of the gel which is employed in the manuscript. Brightness has been adjusted during the processing of the gel.

Supplementary Figure S9

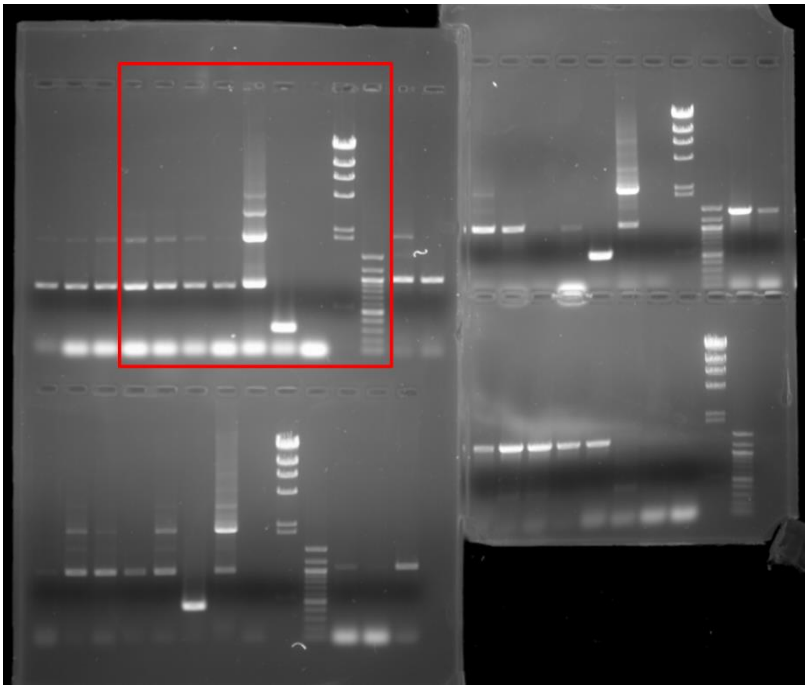

Figure S9. Full length gels of Figure 1e (ii). The red line shows the cropped location of the gel which is employed in the manuscript. Brightness has been adjusted during the processing of the gel.

Supplementary Figure S10

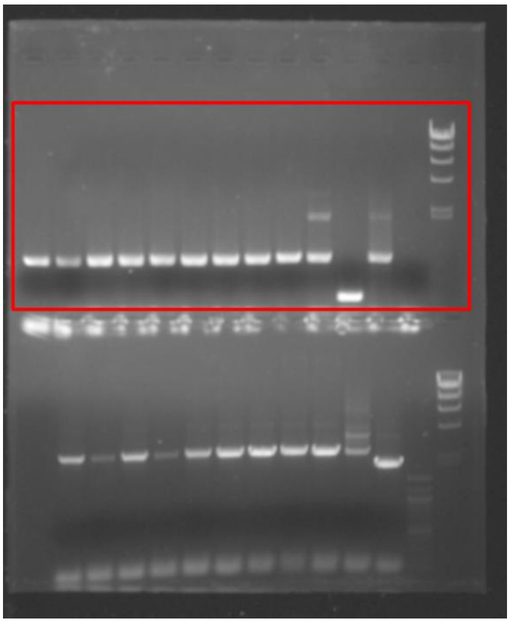

Figure S10. Full length gels of Figure 1f (i). The red line shows the cropped location of the gel which is employed in the manuscript. Brightness has been adjusted during the processing of the gel.

Supplementary Figure S11

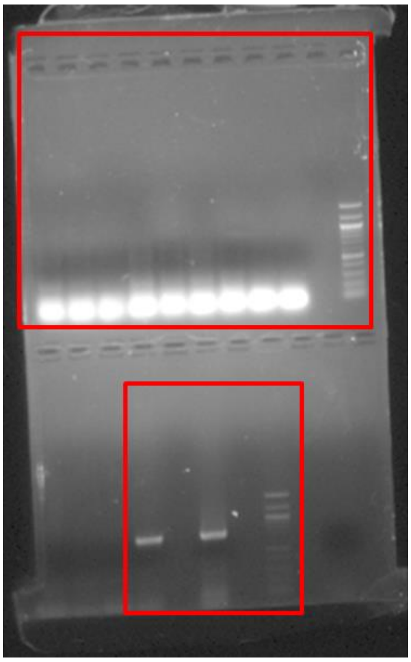

Figure S11. Full length gels of Figure 1f (ii). The red line shows the cropped location of the gel which is employed in the manuscript. Brightness has been adjusted during the processing of the gel.

Supplementary Figure S12

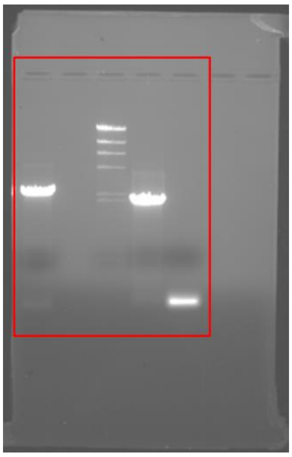

Figure S12. Full length gels of Figure 2b. The red line shows the cropped location of the gel which is employed in the manuscript. Brightness has been adjusted during the processing of the gel.

Supplementary Figure 13

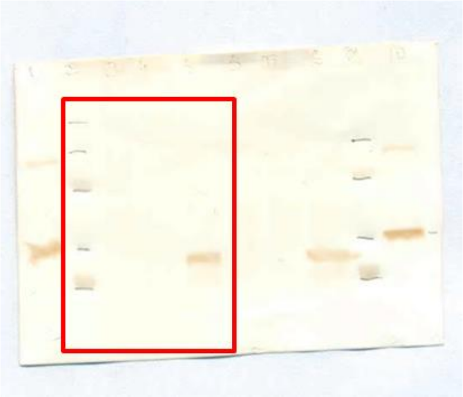

Figure S13. Full length blot of Figure 2c. The red line shows the cropped location of the blot which is employed in the manuscript.

Supplementary Figure 14

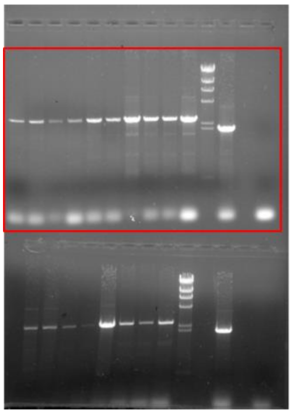

Figure S14. Full length gels of Figure 2d. The red line shows the cropped location of the gel which is employed in the manuscript. Brightness has been adjusted during the processing of the gel.
